# Imaging Neuroinflammation *In Vivo* in a Neuropathic Pain Rat Model with Near-Infrared Fluorescence and ^19^F Magnetic Resonance

**DOI:** 10.1371/journal.pone.0090589

**Published:** 2014-02-28

**Authors:** Kiran Vasudeva, Karl Andersen, Bree Zeyzus-Johns, T. Kevin Hitchens, Sravan Kumar Patel, Anthony Balducci, Jelena M. Janjic, John A. Pollock

**Affiliations:** 1 Biological Sciences, Bayer School of Natural and Environmental Sciences, and Chronic Pain Research Consortium, Duquesne University, Pittsburgh, Pennsylvania, United States of America; 2 NMR Center for Biomedical Research, Carnegie Mellon University, Pittsburgh, Pennsylvania, United States of America; 3 Graduate School of Pharmaceutical Sciences, Mylan School of Pharmacy, and Chronic Pain Research Consortium, Duquesne University, Pittsburgh, Pennsylvania, United States of America; 4 Department of Research and Development, Celsense, Inc., Pittsburgh, Pennsylvania, Unite States of America; Shanghai Jiao Tong University School of Medicine, China

## Abstract

Chronic neuropathic pain following surgery represents a serious worldwide health problem leading to life-long treatment and the possibility of significant disability. In this study, neuropathic pain was modeled using the chronic constriction injury (CCI). The CCI rats exhibit mechanical hypersensitivity (typical neuropathic pain symptom) to mechanical stimulation of the affected paw 11 days post surgery, at a time when sham surgery animals do not exhibit hypersensitivity. Following a similar time course, TRPV1 gene expression appears to rise with the hypersensitivity to mechanical stimulation. Recent studies have shown that immune cells play a role in the development of neuropathic pain. To further explore the relationship between neuropathic pain and immune cells, we hypothesize that the infiltration of immune cells into the affected sciatic nerve can be monitored *in vivo* by molecular imaging. To test this hypothesis, an intravenous injection of a novel perfluorocarbon (PFC) nanoemulsion, which is phagocytosed by inflammatory cells (e.g. monocytes and macrophages), was used in a rat CCI model. The nanoemulsion carries two distinct imaging agents, a near-infrared (NIR) lipophilic fluorescence reporter (DiR) and a ^19^F MRI (magnetic resonance imaging) tracer, PFC. We demonstrate that in live rats, NIR fluorescence is concentrated in the area of the affected sciatic nerve. Furthermore, the ^19^F MRI signal was observed on the sciatic nerve. Histological examination of the CCI sciatic nerve reveals significant infiltration of CD68 positive macrophages. These results demonstrate that the infiltration of immune cells into the sciatic nerve can be visualized in live animals using these methods.

## Introduction

Pain is a public health problem that has deleterious effects on social, mental, physical and economic health of its sufferers [1]. Pain caused by injury or disease associated with the somatosensory nervous system is called neuropathic pain [2]. Recent studies have estimated that at least 6 million Americans suffer from neuropathic pain [3]. Other studies indicate that neuropathic pain is usually associated with nerve inflammation [4], which occurs when leukocytes such as macrophages infiltrate the nerve. Understanding the role of key inflammatory cytokines and immune cells involved in neuropathic pain could lead to a deeper understanding of the signaling mechanisms involved in hypersensitivity as well as better diagnostic and treatment strategies.

The chronic constriction injury (CCI) rat model [5] is a well-characterized example of chronic peripheral neuropathic pain [6]. This model is widely used to simulate Complex Regional Pain Syndrome type II observed in humans (CRPS II, earlier called causalgia) [5]. CRPS II develops when a major peripheral nerve is injured [7] and includes persistent pain and mechanical allodynia [8].

Significant advances have been made in understanding the molecular basis of the diseases and conditions of chronic pain and the hypersensitivity associated with CCI. In many cases, hypersensitivity correlates with changes in the expression of specific ion channels [9]. Among these are cation channel proteins including the Transient Receptor Potential (TRP), a superfamily of receptors [10] that are known for being involved in a variety of sensory processes including nociception and pain [11], [12]. In CCI, there is evidence that seven days after the CCI surgery, TRPV1 protein, but not mRNA expression increases in the Dorsal Root Ganglia (DRG) [13], [14]. Furthermore, neuroinflammation has been associated with CCI-induced mechanical hypersentivity. Clatworthy and coworkers [15] showed that inflammation plays a key role in pathophysiology of neuropathic pain. Furthermore, macrophages have been shown to play a central role in causing the inflammation that contributes to peripheral neuropathies [16]. It has also been demonstrated that Wallerian degeneration occurs in CCI rat model [17]. During Wallerian degeneration, resident macrophages and the Schwann cells are the first cells to respond to the insult and together they initiate the axonal degeneration phase of the distal portion of the damaged nerve. Within the first three days of nerve injury, acute inflammation is evident with the infiltration of neutrophils reaching the site of injury via diapedesis [18]. The peak in neutrophil infiltration is reached within 24 hours of injury. From day 4 to day 14 post injury, hematogenous macrophages infiltrate the injured nerve area, reaching a peak around 1 week post injury [18]. One of the roles of these macrophages is to remove the degenerating nerve tissue debris and to cause inflammation [18]. The infiltration of these immune cells into damaged nerve tissue is an important component of the inflammatory process. However, the role of inflammatory cell infiltration into the site of neuronal injury and how that initiates chronic pain is not well understood. Understanding this process will ultimately lead to the development of new therapeutic agents and better pain management. To study infiltrating immune cells in a rat chronic pain model, we used a novel NIR (near-infrared) labeled perfluoro-15-crown-5 ether (PCE) nanoemulsion [19], shown to preferentially label macrophages in culture and upon intravenous administration. Perfluoropolyethers (PFPEs), including PCE, have been used for ^19^F MRI (magnetic resonance imaging) in multiple animal models [20], [21], [22], [23]. Specifically, PFPE nanoemulsions have become a powerful tool for imaging inflammation in varied disease models, from acute organ rejection to rheumatoid arthritis [24]. PFPE nanoemulsion droplets are taken up from the blood stream by phagocytic cells including monocytes and macrophages and carried by these cells to sites of inflammation. PFPEs in the nanoemulsions are biologically and chemically inert, and are not degraded by the phagocytic cells, which gives PFPEs advantage over other imaging modalities [25]. The ^19^F MR signal remains unchanged during the lifetime of the labeled cell and can be directly correlated to the immune cell number at the site of interest [26]. Further, ^19^F MR signal is specific for the introduced nanoemulsion and provides an unambiguous signature of monocytes and macrophages *in vivo*. In a recent study [19] we reported *in vivo* inflammation imaging demonstrating that NIR and ^19^F MRI can be used as complementary imaging modalities for identifying underlying cellular mechanisms in inflammation.

Previously, aspects of the role of inflammation in neuropathic pain have only been established by *ex vivo* techniques [27], [28], [29], [30]. To the best of our knowledge, there are no reports on visualizing neuroinflammation in live animals using the CCI model of neuropathic pain. While ^19^F MRI has been used previously to image *in vivo* neuroinflammation in the peripheral nervous system [31], we are describing for the first time a novel method to visualize the *in vivo* inflammation associated with nerve injury in the CCI rat. To achieve this, we are using a newly developed nanoemulsion-based dual mode imaging probe [19]. We find that NIR and ^19^F MRI can be used to monitor inflammation at the site of injury on the sciatic nerve. Using multiple *ex vivo* imaging techniques (NIR, ^19^F MRI and histology), we demonstrate that macrophages have infiltrated the sciatic nerve following CCI. We discuss the implication of a noninvasive assessment of neuropathic pain for use in studying pain associated with the hypersensitivity caused by CCI.

## Materials and Methods

### Ethics Statement

This study was carried out in strict accordance with the recommendations in the Guide for the Care and Use of Laboratory Animals of the National Institute of Health, and the Institutional Animal Care and Use Committee (IACUC) at Duquesne University approved the animal protocol (Protocol 1109-10). All surgical procedures were performed under isoflurane anesthesia. Male Sprague-Dawley rats weighing 250–350 g were used in this study (Hilltop Lab Animals, Inc., Scottdale, PA). Rats were maintained on a 12∶12 hour light-dark cycle and were given *ad libitum* access to water and purified chow. Purified chow has been demonstrated to reduce non-specific fluorescence caused by plant materials in basic chow in the LI-COR Bioscience Application Note: *In Vivo* Animal Imaging Diet Consideration (http://biosupport.licor.com/docs/InVivoDiet-Considerations.pdf).

### Chronic Constriction Injury

**Figure 1:**
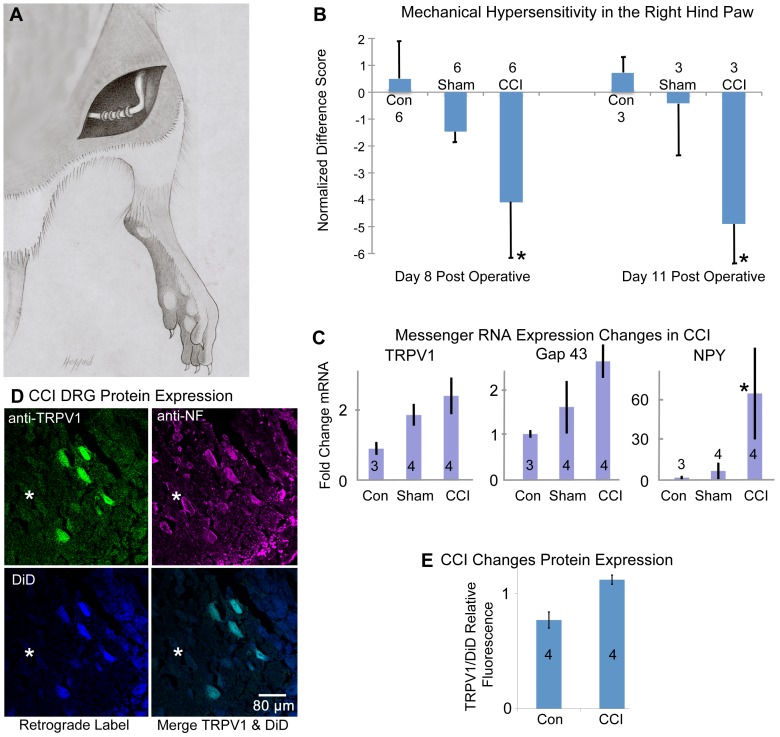
Chronic Constriction Injury (CCI) affects behavior, as well as mRNA and protein expression. **A.** Sketch of the sciatic nerve with the chronic constriction injury sutures in place. **B.** Mechanical hyperentivity in the right hind paw. Bars represent the mean ± SEM normalized 50% withdrawal threshold from calibrated Semmes-Weinstein monofilaments (g) for each of the treatment groups; un-operated control (Con), sham-operated control (Sham), and chronic constriction injury (CCI). The more negative a mean difference score, the lower the pain threshold in the right hind paw. *Significant difference from un-operated control rats (Kruskal- Wallis 1-way ANOVA by ranks; P≤0.05). Un-operated and sham-operated control rats did not differ in normalized pain thresholds at day 8 or day 11 post-operative. Sample size representing each mean indicated on the graph. **C.** Relative Expression of mRNA assessed by qPCR (one step direct priming of mRNA). mRNA extracted from dissected L5 ipsilateral dorsal root ganglia for non-surgical control (Con), sham and CCI were used to compare the expression levels of TRPV1, Growth associated protein 43 (Gap43) and Neuropeptide Y (NPY). All three genes exhibit elevated levels of transcript expression in the CCI condition that were significantly different from control, indicating peripheral nerve trauma and neuropathic pain. For TRPV1, these data show that the CCI-treatment group was significantly different from the control groups (p<0.05). The CCI-treatment group was not significantly different from the sham group (p>0.05). For GAP43, these data show that the CCI-treatment group was significantly different from the control groups (p<0.05). The sham-treatment group was not significantly different from the control group (p>0.05). For NPY, these data show that the CCI-treatment group was significantly different from both the sham-treatment group and the control group (p<0.001). Sample size representing each mean indicated on the graph. **D.** DiD retrogradely labeled neurons from the right footpad of a CCI surgical animal. Cell bodies in the L5 doral root ganglia were processed for multi-stain confocal microscopy with anti-TRPV1 and anti-Neurofilament antibodies. The results reveal that some neurons co-label with TRPV1 and DiD, while other neurons identified with anti-Neurofilament antibody (*) lack both DiD and TRPV1. Scale Bar = 80 µm. **E.** Using Leica confocal software, the relative fluorescence of TRPV1 to DiD in co-expressing cells for representative CCI and comparably stained tissue sections from control DRG is shown. Sample size representing each mean indicated on the graph. TRPV1 protein expression is elevated in the CCI condition.

The chronic constriction injury (CCI) method developed by Bennett and Xie [5] was used to induce neuropathic pain in the sciatic nerve of rats (Figure 1A). Animals were divided into three groups; CCI, sham and naïve un-operated control. Briefly, under general anesthesia, the common sciatic nerve of the CCI animals was exposed by making an incision through the skin and carefully separating the muscles in the mid thigh region of right hind limb. Four ligatures were tied around the common sciatic nerve using 4-0 chromic gut suture. Care was taken to ensure that the ligatures were secured in place without restricting epineurial blood flow. The muscle layer was closed using 4-0 chromic gut suture followed by skin closure using 9 mm stainless steel wound clips. Sham rats underwent identical surgery except that the ligatures were not tied around the nerve. Naïve control rats did not undergo any surgical procedures and are referred to as un-operated controls.

### Behavioral Testing

On the day of surgery, prior to commencement of any surgical procedures, CCI, sham and control rats underwent baseline assessment for mechanical sensitivity. Behavioral testing was repeated on day 8 and day 11 post surgery. Following previously established methods, mechanical pain threshold was assessed by applying calibrated Semmes-Weinstein monofilaments to the plantar surface of both the left and right hind paw, specifically in the cutaneous region that is innervated exclusively by the sciatic nerve [32], [33].

A linear regression was used to estimate each rat’s 50% withdrawal threshold. As previously described [32], rats were placed on a metal grid and covered with a Plexiglas cover. The lowest-caliber filament (0.41 g) was pushed onto the plantar surface of the right hind paw 5 times until the filament bent; 2 to 3 seconds separated each push. Five minutes later, the left paw of the same rat was similarly assessed. Not less than 5 minutes after the left paw was assessed, the entire procedure was repeated. In this way, the right and left paws were probed with the 0.41 g filament 10 times, and the number of withdrawals was recorded. In ascending order, each filament was so tested until the rat withdrew from all 10 pushes of a single-sized filament or until the largest-caliber filament was tested.

To estimate the force from which the rat withdrew 50% of the time, force (in grams) was regressed on withdrawal frequency for all 5 filaments by using simple linear regression. The force at which the rat withdrew a paw on 5 of 10 pushes (50% withdrawal threshold) was predicted by using the regression equation. If the 50% withdrawal threshold so calculated was greater than 15.13 g, the highest-caliber filament used (15.13 g) was recorded as the gram force. If the rat failed to respond to any of the filaments, 15.13 g was recorded as the 50% withdrawal threshold. The same investigator always performed this test. Threshold levels were then expressed as a difference score between the pain threshold for the right and left hind paws. These values were normalized to differences that existed between the 2 paws at baseline. Statistics were performed using SPSS V12.0 software. We observed significant difference between the CCI condition and the un-operated control rats (Kruskal- Wallis 1-way ANOVA by ranks; P≤0.05). All behavioral testing was done by experimenter blinded to the surgical treatment. Rats used for the detailed behavioral testing consisted of a separate cohort from those used for the DRG analysis and *in vivo* imaging. All CCI animals were observed to exhibit guarding, paw licking and or paw lifting behaviors by day 8 post-surgery. However, only normal grooming behaviors were seen in the sham and un-operated controls.

### Dorsal Root Ganglia Dissection

Nine days post surgery, bilateral L3, L4 and L5 DRG were dissected from CCI rats via laminectomy. These ganglia were chosen because neurons within them provide the sensory component of the sciatic nerve [34], [35]. Ganglia were rapidly removed and stored in RNA*later* solution (Qiagen) for later use. Rats were sacrificed after completion of DRG extraction.

### RNA Isolation and qPCR Analysis

Quantitative PCR (qPCR) and the comparative cycle threshold (CT) method [36] were used to determine if the CCI altered the mRNA expression of TRPV1, Neuropeptide Y (NPY) and Growth-associated Protein 43 (Gap43) relative to Glyceraldehyde 3-phosphate dehydrogenase (GAPDH) in L5 DRG 12 days post surgery (see Table S1 for primers). GAPDH, an important enzyme in glycolysis, serves as a control transcript for comparisons to transcripts from other genes expressed within the same tissue [37]. Tissue samples were removed from the RNA*later* solution, processed over QiaShredder columns and RNA was extracted using the RNeasy kit (Qiagen, Valencia, CA). To prevent any genomic DNA contamination, each RNA sample was treated with DNase I (Invitrogen, Grand Island, NY).

Using RNA as the template, one step qPCR was performed with gene specific primers. Reverse-transcription was performed on the StepOnePlus machine using the *Power* SYBR Green RNA-to-CT *1-Step* Kit (Applied Biosystems). RNA extracted from each rat was analyzed separately in triplicate. Analysis of amplified cDNA products by agarose gel and subsequent DNA sequencing confirmed the identity of the intended PCR product. All statistical tests were performed using SPSS software. Assumptions of ANOVA were met as indicated by Mauchly’s Test of Sphericity and Levine’s Test for Equality of Variances. Significance of the F statistic was defined as *p*≤0.05. TRPV1 mRNA levels in right (ipsilateral to the nerve injury) DRG of CCI rats was compared to right DRG of control (pooled) rats by performing a 1-Way ANOVA with treatment as the between-subjects factor.

### Immunohistochemistry and Confocal Microscopy

Dorsal root ganglia from control and CCI rats, were used for immunohistochemistry to explore the protein expression levels of TRPV1. Under general anesthesia, prior to any surgical procedures, the CCI and control rats were injected with DiD Dye (V-22887, Invitrogen, Carlsbad, CA) in the plantar region of the right hindpaw. DiD is a lipophilic retrograde tracer that will stain all neuronal cells innervating the hindpaw. Post-treatment, all DRGs were removed and immediately fixed in 4% paraformaldehyde 1X Phosphate Buffer Saline (PBS) solution. The tissue samples were cryoprotected in 30% sucrose 1X PBS solution for 4 hours. After four hours of incubation, 1/3 of the cryoprotectant solution was replaced with Optimal Cutting Temperature (OCT) compound (Sakura-Finetek, Torrance, CA) and incubated on a rocking shaker overnight. After overnight incubation, samples were embedded in OCT and 20 µm thick sections were made on a cryostat. Sections were postfixed in 4% paraformaldehyde solution 1X PBS, permeabilized with 0.3% Triton X-100 solution and stained with primary and secondary antibodies. The following primary antibodies were used: 1∶50 mouse anti-rat Neurofilament-M (Neuromics, Edina, MN), 1∶50 rabbit anti-rat TRPV1 (Santa Cruz Biotechnology, Dallas, TX). The sections were then washed and incubated overnight with anti-mouse secondary antibodies conjugated to Alexa-546 (Invitrogen, Carlsbad, CA), and anti-rabbit-FITC (Santa Cruz Biotechnology, Dallas, TX), diluted 1∶100 in PBS. Sections were washed in 1X PBS and mounted in Prolong-Gold (Invitrogen, Carlsbad, CA). Relative expression of TRPV1 was determined by comparing the fluorescence intensity for TRPV1 to the fluorescence intensity of DiD, whose fluorescence intensity is not affected by the CCI surgery. This was done for labeled neurons from naïve control DRG and CCI DRG using the Leica TCS SP2 software version 2.3. For a double-labeled cell, the overall TRPV1 mean amplitude was divided by the overall mean DiD amplitude. The results of an unpaired t-test showed t = −6.45, sdev = 0.758E-01 degrees of freedom = 6. The probability of this result, assuming the null hypothesis, is 0.0007. An experimenter blind to treatment did staining and quantification of immunohistochemistry.

In separate immunohistochemical experiments using comparable protocols, the recovered control, sham and CCI sciatic nerves were prepared for examination using mouse anti rat CD68 antibody (MCA341R, AbD Serotech, Raleigh, NC) and Alexa fluor 488 donkey anti mouse secondary antibody (A-21202, Invitrogen, Carlsbad, CA) to assess the presence of macrophages that infiltrate the nerve.

Cellular uptake of nanoemulsion was performed in the mouse leukemic monocyte macrophage cell line RAW 264.7 (ATCC, Manassas VA) as described by Patel *et al* [38]. Approximately, 0.3 million cells were seeded on cover slips in 6-well plates in 2 mL culture medium per well and grown as adherent macrophages. After 48 h incubation at 37°C and 5% CO_2_, medium was aspirated and cells washed with 1X PBS. Cells were exposed to 2 mL of either fresh culture medium alone or dispersed nanoemulsion containing medium (10 µL/mL). Following 5 h incubation, 1 mL medium was aspirated and cells fixed with 1 mL 4% paraformaldehyde in 1X PBS. After 30 minutes at room temperature, the cells were washed with 1X PBS three times. Each coverslip with and without nanoemulsion labeled cells was transferred to glass slides containing a drop of ProLong Gold mounting medium. The nanoemulsion labeled with DiR (Invitrogen, Carlsbad, CA) was detected with 633 nm laser excitation and emission window of 700 nm to 850 nm. Confocal microscopy was performed on a Leica SP2 spectral Laser Scanning Confocal microscope. A transmission DIC image was acquired with each confocal scan.

### NIR Labeled PFPE Nanoemulsion

NIR labeled PFPE Nanoemulsion (V-Sense DM NIR) was produced and provided by Celsense Inc (Pittsburgh, PA, USA) and was used without further modifications. Previous studies have demonstrated that the nanoemulsion droplets are on the order of 180–200 nm in size [19].

### Live Animal Imaging (NIR)

To follow the localization of emulsion labeled circulating monocytes (monocytes in the blood, macrophages in tissue), NIR imaging of the fluorescent label in live rats was performed on a LiCOR Pearl Impulse (LI-COR Biosciences, Lincoln, NE) small animal imaging system. For the live animal imaging, rats were fed purified chow (D10012G Research Diets, Inc. New Brunswick, NJ), which helps to reduce non-specific fluorescence in the abdominal and thoracic region of the animal caused by certain foods. Tail-vein injection of 300 µL of V-Sense DM nanoemulsion was performed 8 days after surgery under general anesthesia. Within minutes, the injected animals were imaged in the LiCOR imager to establish that the intravenous injection was successful and that the nanoemulsion was not leaking subcutaneously within the tail. On day 11 post surgery, maximum neuroinflammation was predicted based on previous results, and another set of NIR imaging was performed under general anesthesia (xylazine, 7 mg/kg/ketamine, 80 mg/kg) given intraperitoneally. Simultaneous image acquisition of white light (body view) and 785 nm excitation for 820 nm emission were merged and processed in the LiCOR Pearl Impulse Software (version 2.0) with linked look-up-tables (LUT). Relative fluorescence was measured from a region of the same size over the sciatic nerve for both left and right legs for the naïve, sham and CCI conditions. This involved identifying a region of interest (ROI) around the fluorescent signal evident in the CCI. That ROI perimeter was then copied to the other legs for a given experiment. In this way, nearly the same number of pixels of area for the signal is compared within a given paired experiment. The ROI images for each rat (Figure S1) and the tabulation of relative fluorescence from the LiCOR Pearl Impulse software (Table S2) are shown in Supporting Information. Prism software version 5.0 was used to perform analysis of variance (ANOVA) for the entire set of conditions revealing a p value<0.0001 (Table S3). Furthermore, a t-test specifically comparing the sham right leg to the CCI right leg reveals a p value<0.0001. Dissected sciatic nerves were similarly imaged for NIR fluorescence on a flatbed LiCOR Odyssey Infrared Imaging system. An experimenter blinded to experimental conditions did the analysis of NIR Pearl images.

### MRI (^1^H & ^19^F) Methods

^1^H and ^19^F MRI was performed on rat legs using a Bruker Biospec Avance III 7-T/21-cm system (Bruker Biospin MRI, Billerica MA) and a 35-mm (^1^H/^19^F) double resonance birdcage coil (Rapid MR International, Columbus OH). Following perfusion fixation, dissected legs were placed in a 50 mL conical tube and positioned in the magnet. Following pilot scans, the anatomical images were collected using a RARE (Rapid Acquisition with Relaxation Enhancement) sequence with both axial and a double oblique (parallel to the femur/tibia plane) coronal views. The following acquisition parameters were used: TR/TE 4000/7 ms, RARE Factor = 8, NA = 4, 6×6 cm FOV, 256×256 matrix, 2-mm slice thickness, and 22 or 14 contiguous slices for axial or coronal views, respectively. ^19^F MRI was performed at 282 MHz with the same slice geometry and the following parameters: TR/TE 3000/7 ms, RARE Factor = 4, 6×6 cm FOV, 128×128 matrix, and NA = 256 or 384 for axial or coronal views, respectively. Since the resonance frequencies were centered precisely on the water and the perfluorocarbon resonances, ^19^F and ^1^H anatomical images were co-registered without additional image processing.

^19^F NMR spectra for the PFPE nanoemulsion in the dissected sciatic nerves were acquired following standard protocols with trifluoroacetic acid (TFA) as the internal standard [38]. Nerves were inserted into borosilicate NMR tubes (5 mm diameter) and spectra recorded (Bruker, 470 MHz). ^19^F NMR peak around −92.5 ppm corresponding to fluorine nuclei was measured with TFA (set at −76.0 ppm) as reference (Figure S2).

## Results

In this study, we demonstrate that CCI rats exhibit symptoms of mechanical hypersensitivity, which is a hallmark of chronic pain. We also show that dual mode PFPE nanoemulsion can be used to reveal the focal point of neuroinflammation by both NIR imaging and ^19^F MRI. Furthermore, we demonstrate that the NIR and ^19^F signal is arising from CD68 positive macrophage cells carrying the nanoemulsion that infiltrate the CCI injured nerve. Due to anatomical barriers for free nanoemulsion delivery to the site of injury and considering the supporting histological findings, the NIR and ^19^F MRI signal shown arise from infiltrating cells.

Figure 1B illustrates that in the CCI rat, mechanical hypersensitivity is evident 11 days post surgery. Guarding behavior and other behaviors that are indicators of increased sensitivity were evident in all CCI animals. We also demonstrate that under these conditions, the mRNA expression levels of TRPV1, NPY and Gap43 are elevated on day 12 post surgery (Figure 1C). For TRPV1, the mean fold change values for the CCI-treatment, sham-treatment, and control groups were 2.60±0.352, 2.06±0.30, and 1.11±0.18, respectively. These data show that the CCI-treatment group was significantly different from the control groups (p<0.05). The CCI-treatment group was not significantly different from the sham group (p>0.05). For GAP43, the mean fold-change values for the CCI-treatment, sham- treatment, and control groups were 2.42±0.32, 1.52±0.51, and 1.00±0.07, respectively. These data show that the CCI-treatment group was significantly different from the sham-treatment and control groups (p<0.05). The sham-treatment group was not significantly different from the control group (p>0.05). For NPY, the mean fold change values for the CCI-treatment, sham-treatment, and control groups of 64.20±33.79, 6.49±6.21, and 1.44±1.34, respectively. These data show that the CCI-treatment group was significantly different from both the sham-treatment group and the control group (p<0.001).

To demonstrate that the expression of the TRPV1 protein is similarly elevated in the L5 dorsal root ganglia of CCI animals as compared to control, we analyzed tissue sections with antibodies against TRPV1 and Neurofilament-M (a neuronal marker). Additionally, the plantar region of the right rear paw of each rat was injected with DiD, a retrograde fluorescent dye that identifies the neurons innervating that specific region of the foot. Not all large diameter neurons in the dorsal root ganglia were positive for the DiD retrograde tracer since only a discrete number of neurons reach that region of the footpad. However, several DiD positive neurons are found and of those, some co-express TRPV1 in both control and CCI tissue. In the control, the immunofluorescent ratio of TRPV1/DiD is 0.787 indicating that under these conditions the DiD signal is brighter than the TRPV1 signal. However, in the CCI the TRPV1/DiD ratio is 1.12 (Figure 1E), indicative of elevated TRPV1 protein expression in these neurons of the dorsal root ganglion.

**Figure 2:**
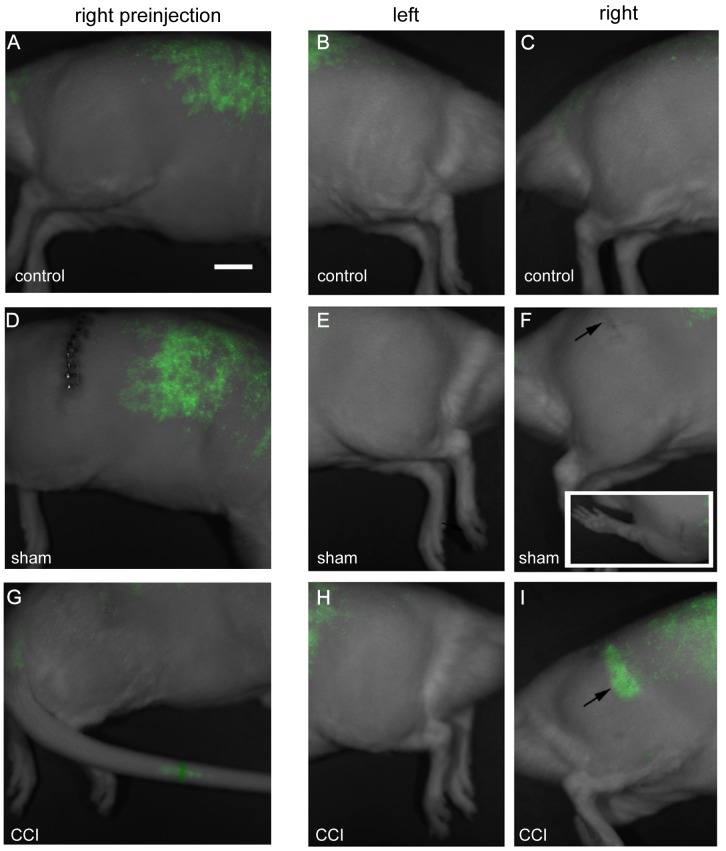
Near Infrared imaging (NIR) of live rats merged with white light images of their body, 2 days post intravenous injection of labeled emulsion on day 11 post surgery. All of these images were acquired on the LiCOR Pearl Live animal imager and processed in the same experiment with linked look-up tables. The control (A, B, C) and sham (D, E, F) exhibit only auto fluorescence over the thoracolumbar region, which is evident prior to injection (A, D, G). The inset in F exhibits a dorsal view of the wound, which lacks any fluorescent signal. The CCI affected right leg (I) exhibits a clear band of fluorescent signal aligned with the position of the sciatic nerve (arrow), which runs parallel to the femur. No signal is evident on the CCI animal’s unaffected left leg (H). Note – supplemental data (Figure S3) of a comparable experiment reveals only autofluoresence in sham and naïve controls, while only the CCI exhibits extensive signal along the length of the thigh, parallel to the position of the right sciatic nerve. Bar = 1 cm.

**Figure 3:**
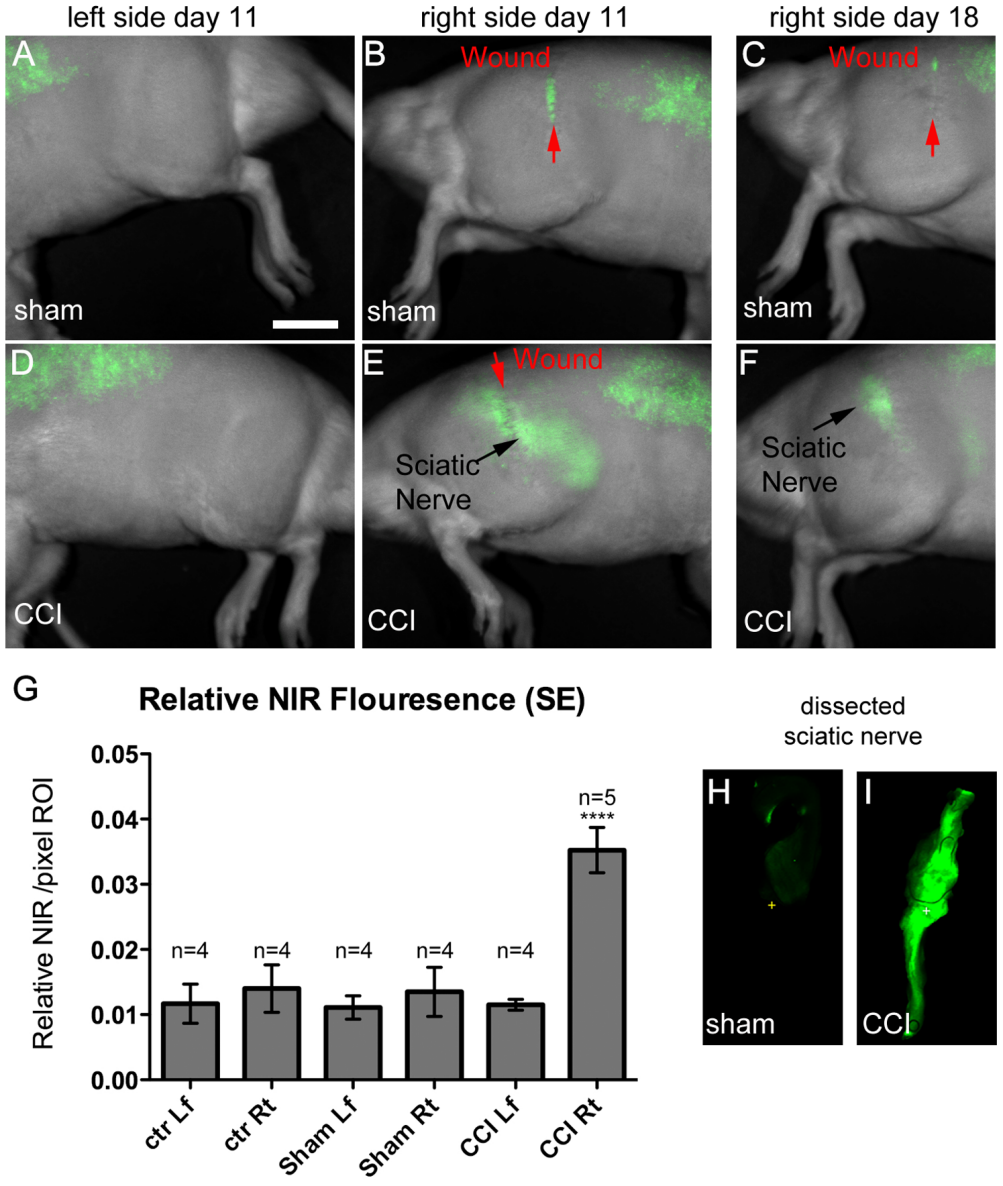
Near Infrared imaging (NIR) of live rats merged with white light images of their body, 2 days post intravenous injection of labeled emulsion on day 11 post surgery compared to day 18 post surgery. All of these images were acquired on the LiCOR Pearl Live animal imager and processed in the same experiment with linked look-up tables. Bar = 1 cm. The sham surgical control (**A**, **B**, **C**) exhibits auto fluorescence over the thoracolumbar region, which is also evident prior to injection. In the sham animal, a superficial fluorescence signal is detected precisely on the surgical wound. That surgical wound signal is dramatically reduced by day 18 (**C**) in the same sham animal. The CCI experimental animal (**D**, **E**, **F**) exhibits auto fluorescence over the thoracolumbar region, which is also evident prior to injection. The CCI affected right leg (**E**) exhibits a clear band of fluorescent signal aligned with the position of the sciatic nerve (black arrow) along the entire length of the thigh. No signal is evident on the CCI animal’s unaffected left leg (**D**). On day 18, the signal in the Sham attributed to the wound has nearly completely resolved and no signal is apparent along the sciatic nerve (C). The CCI sciatic nerve associated signal persists in at day 18 (F). Bar = 1 cm. **G**. The relative fluorescence of a region over the sciatic nerve is compared. For each condition ROI measurements were made (Figure S1, Table S2) for the 4 sets of data giving a sample number of 4 to 5. The results of the ANOVA test (Table S3) show that the relative fluorescence in the CCI right is significantly different from the remainder of the samples with a p value<0.0001. Separately, a t-test analysis of the Sham right versus the CCI right revealed a p value of <0.0001. Sciatic nerve dissected from comparable animals imaged simultaneously in the same field of view for NIR fluorescence on the LiCOR Odessey Flatbed Imager (H, I). The CCI sciatic nerve (I) exhibits a strong fluorescent signal in comparison to the sham sciatic nerve (H).

Figure 2 reveals that after the intravenous injection of the NIR labeled PFPE nanoemulsion, CCI injured rats exhibits NIR fluorescence that is focally localized parallel to the thigh (Figure 2I) at 11 days post CCI. This is in contrast to the autofluorescence evident in sham (Figure 2F) and control (Figure 2C) in the thoracolumbar regions, which is similar to that seen prior to the nanoemulsion injection (Figure 2 A, D, G). In a separate experiment, the surgical wound was occasionally found to exhibit low-level NIR fluorescence (Figure 3 B,C). This indicated that there was some superficial inflammation at the surgical wound as compared to the deeper sciatic nerve signal, which exhibits more scattering along the length of the sciatic nerve (Figure 3 E,F). Using the Li-COR Pearl Impulse software version 2.0 image analysis tools, we identified regions of interest (ROI) over the affected sciatic nerve for the CCI, sham and control animals (Figure S1 and Table S2). By averaging the normalized fluorescence (total relative fluorescence/total area) of the right and left legs, we demonstrate in Figure 3 G, that the CCI fluorescence is significantly brighter than the controls. ANOVA analysis of the entire data set comparing the relative fluorescence detected in the naïve right and left legs, the sham right and left legs and the CCI right and left legs reveals a difference for the CCI affected right leg with a p value<0.0001 (Table S3). A t test specifically comparing the affected CCI right leg to the sham surgical right leg reveals a significant difference with a p value<0.0001 (Table S3). However, given that NIR imaging cannot unequivocally provide spatial localization of the fluorescence, we continued the analysis by assessing the fluorescence of the dissected sciatic nerve from the sham as compared to the CCI animal (Figure 3 H,I). With CCI and sham nerves lying side-by-side and imaged simultaneously on the flatbed Li-COR Odyssey imaging system, only the CCI shows positive signal.

In preparation of the MRI analysis of the tissues, we assessed the NMR spectra of the dissected sciatic nerves to ascertain that the only fluorine signature to be detected was arising from the nanoemulsion and not any contribution that may come from natural sources or from anesthesia. The NMR spectra (Figure S2) show that the only detectable fluorine in the sciatic nerve arises from the PFPE nanoemulsion.

**Figure 4:**
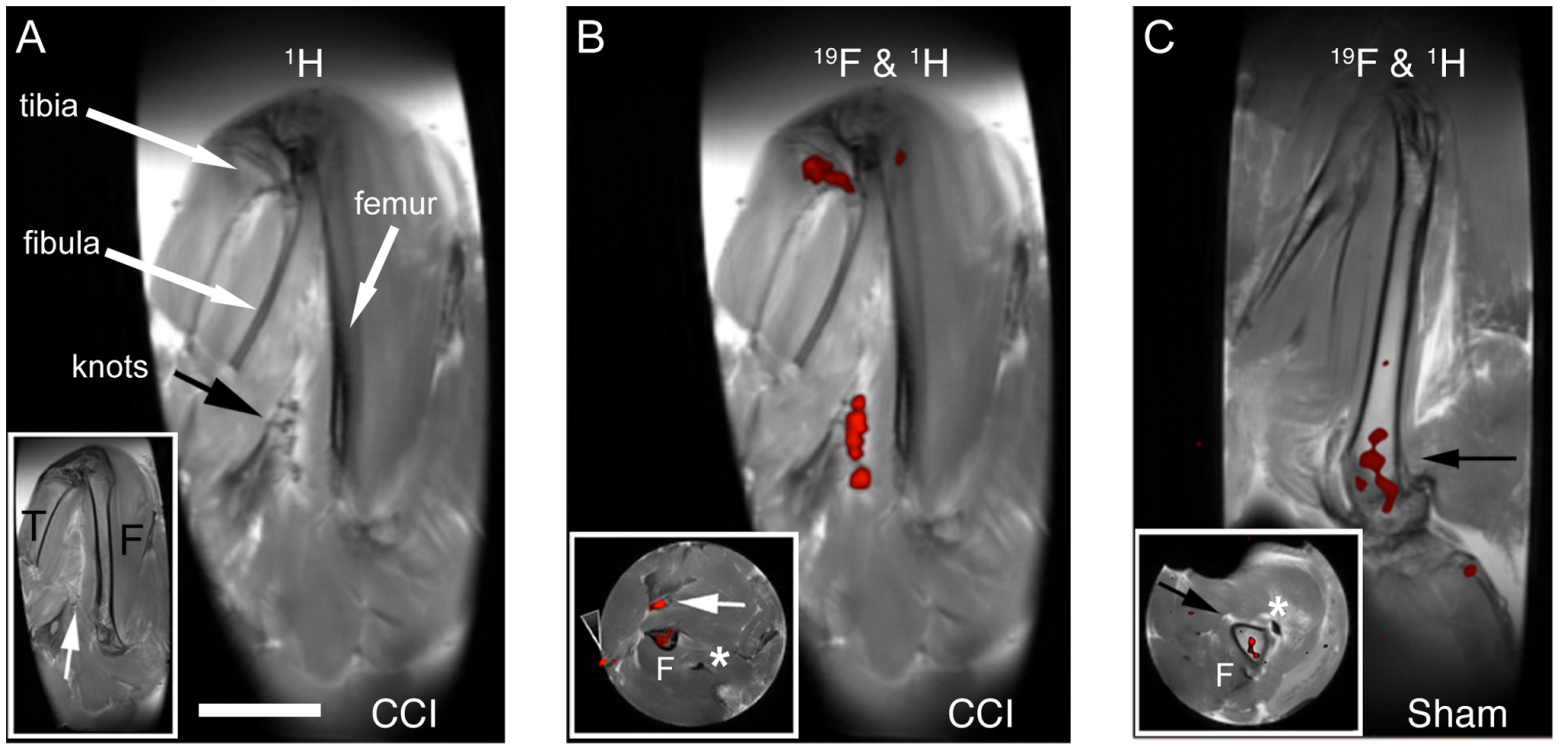
^1^H and ^19^F MRI of the hind limbs for the rats shown in Figure 2F and 2I. Panel **A** shows an anatomical (^1^H) image of the leg for the animal shown in 2I. The black arrow indicates the sutures used for the chronic constriction injury. The long white arrows indicate the position of the tibia, fibula and femur. The inset image shows the adjacent image slice, giving a clearer view of the tibia (T) and the femur (F). The white arrow in inset panel A points to the sciatic nerve, which appears as a white line. Panel **B** shows a composite ^1^H/^19^F image. The ^19^F image is rendered in pseudo color (red) and is overlaid on the anatomical image shown in **A**. A significant amount of ^19^F signal is observed at the sutured sciatic nerve. A small amount of fluorine signal is also evident at the head of the tibia and slight in the femur. The inset shows an axial view of the thigh revealing the femur in cross section. A small amount of fluorine signal is observed at the surface aligning with the surgical wound, where minor superficial inflammation is still evident (arrow head). The (F) indicates the femur in cross sections with a low level of ^19^F signal. The white arrow indicates the sciatic nerve with ^19^F signal. The asterisk (*) is near the femoral artery. Panel **C** shows a ^1^H/^19^F composite image of the sham right leg from the animal shown in Figure 2F. No fluorine signal was observed at the sciatic nerve, however a weak signal was observed in the femur, presumably in the bone marrow (arrow). The inset shows an axial view at the level of the arrow near the head of the femur (F) with ^19^F signal in the bone marrow. The black arrow points to the femur and the asterisk (*) is near the femoral artery. Bar = 1 cm.

Analysis of the same right legs as those shown in Figure 2F for the Sham and Figure 2I for the CCI condition by proton and ^19^F MRI show that the PFPE signal is coincident with the ligation of the sciatic nerve (Figure 4). Figure 4A shows that the four suture knots associated with the constriction injury are visible by proton MRI along the sciatic nerve, which is running parallel to the femur. The sciatic nerve (white arrow inset), tibia (T) and femur (F) are clearly visible in the adjacent image slice (Figure 4A – inset). Fluorine signal is evident along the sciatic nerve in the region of the sutures (Figure 4B) as well as in the bone marrow of the proximal head of the tibia. Viewed in cross section (Figure 4B – inset), ^19^F signal is evident in the sciatic nerve, the femur as well as a slight signal where some minor inflammation persists at the site of the surgical wound. The clips had been removed on day 7 post-surgery. In the sham, a lower level of ^19^F signal is evident only in the bone marrow near the head of the femur (Figure 4C). A comparable experiment revealed PFPE nanoemulsion labeled inflammation in the region of the CCI sciatic nerve (Figure S3C) as compared to control and sham.

**Figure 5:**
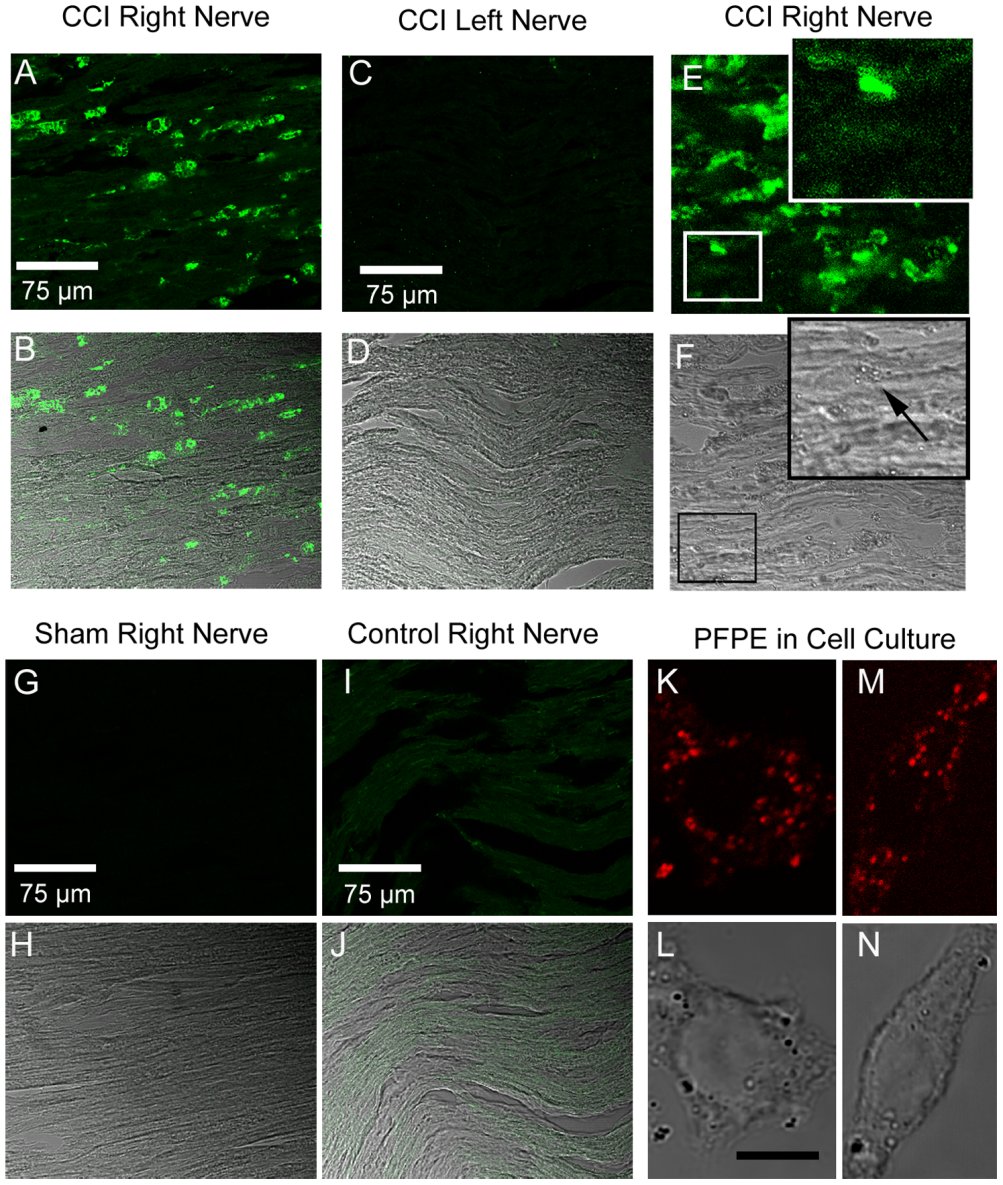
Cryosection of sciatic nerve. **A**,**B** show the affected right sciatic nerve from the CCI leg shown in Figure 2I stained with anti-CD68 antibody to reveal the presence of macrophages infiltrating the nerve. **C**, **D** CD68 positive cells are not present in the left leg (contralateral to surgery Figure 2H) of the same CCI animal. **E**,**F** show a nerve from a separate CCI animal that also exhibits infiltration of CD68 positive cells. The boxed area is enlarged to reveal the granular cytoplasm (black arrow) of the macrophages, indicative of the presence of the nanoemulsion. Sham surgical sciatic nerve and non-surgical control sciatic nerve do not exhibit any CD68 positive cells (**G**,**H**) and (**I**,**J**). The fluorescent images A, C, G, and I were all acquired at the same sitting with the exact same image acquisition parameters. Macrophages grown in cell culture take up the nanoemulsion, exhibited as particles evident by both confocal fluorescence emissions 700–850 nm of NIR label (DiR) (**K**, **M**) as well as transmitted light (**L**, **N**).

To confirm that the PFPE signal is localized to the site of neuroinflammation byinfiltration of macrophages into the actual nerve, we sectioned the recovered sciatic nerve from the CCI right leg and the left leg of the same animal shown in Figure 2H, I and Figure 4A, B and stained the nerve with anti-CD68 antibody (Figure 5A–D). Similarly, the sciatic nerves from the un-operated control and the sham were also recovered for analysis (Figure 5 G–J). The results clearly demonstrate that macrophages have infiltrated into the CCI nerve, but not any of the controls. In duplicate experiments, examination of the macrophages revealed that they exhibit granular cytoplasm (Figure 5E, F). The nanoemulsion droplets in these cells are consistent with the nature of nanoemulsion droplets detected in macrophages grown in cell culture, which measure at the limit of spatial resolution for the light microscope with a size of 200–300 nm (Figure 5 K, L, M, N), which is consistent with the analytic size measurement of 180–200 nm in diameter [19].

## Discussion

Here we report the first live imaging of neuroinflamation in the CCI neuropathic pain model with NIR and ^19^F labeled nanoemulsion taken up by circulating monocytes that infiltrate into the damaged sciatic nerve and present as macrophages.

Chronic neuropathy in the CCI rat model leads to mechanical hypersensitivity [39]. Figure 1B demonstrates significant induction of mechanical hypersensitivity in ipsilateral hindpaw of CCI rat. We note that the role of TRPV1 as a nociceptor has been widely documented in a variety of acute, chronic, and neuropathic pain models and diseases such as diabetic neuropathy, chronic pancreatitis, cancer pain, osteoarthiritis, and gastrointestinal diseases [40], [41], [42], [43]. NPY is a peptide neurotransmitter released by primary afferent neurons located in the dorsal root ganglia and received by neurons located in the substantia gelatinosa region of the spinal cord [44]. In animal models of neuropathic pain, NPY has been shown to be upregulated [45], [46], and can be used as an indicator that trauma to the nerve has occurred. During development, Gap43 is a growth-associated protein that is highly expressed in the growth cones of elongating axons. Its expression decreases after the target has been innervated [47]. In addition to its developmental role, Gap43 is also differently expressed following neuronal tissue damage or inflammation, which has been demonstrated in several animal neuropathy models [47], [48], [49] and can be used to verify that trauma has occurred in peripheral nerve tissue.

We have demonstrated that in the CCI pain model, changes in mRNA expression levels of TRPV1 as well as NPY and Gap43 are evident in lumbar level five dorsal root ganglia using one-step qPCR analysis (Figure 1C). TRPV1 is known to play a role in inflammatory as well as neuropathic pain models [50]. The increased expression of NPY, a neurotransmitter, is known to play a role in modulating both innate and adaptive immune response [51], including modulating the phagocytic uptake by neutrophils and macrophages [52]. Furthermore, NPY has been shown to modulate neuronal signaling in acute pain, neuropathic pain and inflammatory animal pain models and induces anti-nociceptive effects [44]. The increase in NPY expression provides indirect evidence to support the interpretation that nerve trauma has been induced on the sciatic nerve. We find that mRNA expression of TRPV1 was increased in the ipsilateral dorsal root ganglia level 5 as compared to the control rat (Figure 1C). Furthermore, neurons in the dorsal root ganglia that are retrograde labeled with DiD from the plantar foot pad injection exhibit a relative increase in TRPV1 protein expression in the CCI condition as compared to un-operated control rats (Figure 1D, E). Collectively, these results demonstrate that CCI is inducing a hypersensitivity associated with changes in gene expression for the nerves of the affected DRG and sciatic nerve.

We also reported the *in vivo* imaging of neuroinflammation in the CCI sciatic nerve using dual mode (NIR and ^19^F MRI) nanoemulsion. Both resident and hematogenous immune cells are involved in causing neuroinflammation at the site of injury in CCI rat model [18]. The infiltration of immune cells into the site of injury results in the release of inflammatory mediators, which then cause changes in the gene expression profile of the affected neurons and leads to peripheral sensitization [53]. *In vivo* imaging of granulomatous inflammation has been previously reported via labeling of macrophages with fluorescent NIR dye [54]. However, optical imaging is compromised due to the absorption and scattering of both the excitation and emitted light [55]. ^19^F MRI has an advantage in that it is not limited by depth and has no background noise in tissues [56]. ^19^F MRI has also been successfully used for *in vivo* imaging and quantification of inflammation in various animal disease models [20], [31], [57]. Previously, we demonstrated *in vitro* phagocytic uptake of a dual labeled nanoemulsion by macrophages in culture [38]. Here, we again demonstrate that a perfluorocarbon nanoemulsion containing both NIR dye and ^19^F [19] is taken up by macrophages in culture as an assessment of toxicity and uptake as previously measured [19], [38], which gave us confidence that we could achieve adequate sensitivity *in vivo* by both imaging modalities (Figure 5 K–N). This nanoemulsion is then used in order that we might achieve maximum sensitivity and specificity *in vivo*, in the analysis of neuroinflammation in CCI.

On day 8 post surgery, an intravenous injection of the nanoemulsion was given to the rats followed immediately by NIR fluorescence assessment to ensure that the injection was not inadvertently released subcutaneously. In the bloodstream, the phagocytic cells evidently engulf these nanoemulsion droplets through a still undetermined mechanism. These labeled immune cells naturally migrate to the site of injury to participate in the inflammatory response. NIR fluorescence emission from the labeling was observed only on the ipsilateral side of the CCI rat overlying the site of sciatic nerve ligation (Figure 2I and Figure 3E, F, I). Fluorescence was absent on hind limbs of sham rat, control rat and the contralateral hind limb of CCI rat (Figure 2 B, C, E, F, Hand Figure 3 A–D). NIR imaging was used to reveal the accumulation of immune cells at the site of sciatic nerve injury in live animals. Macrophages are the primary immune cells that phagocytose perfluorocarbon nanoemulsion droplets and they represent the majority of all the labeled cells [20]. The results from our *in vivo* NIR imaging suggest that the fluorescence signal is associated with neuroinflammation at the site of sciatic nerve injury in CCI rats. To confirm that the observed fluorescence originated in the sciatic nerve, *ex vivo* NIR imaging of the recovered injured sciatic nerves was performed. Fluorescence emission for NIR dye was observed only in the CCI ipsilateral sciatic nerve and not the ipsilateral nerve of the sham animal (Figure 3 H,I). The results complement the findings from the *in vivo* imaging and show that a fluorescence signal is present in the dissected nerve from the CCI rat, which is indicative of the accumulation of labeled macrophages.

To confirm that the fluorescence signal at the site of CCI injury was due to accumulation of nanoemulsion labeled macrophages, highly sensitive^19^F NMR spectroscopy and ^19^F MRI were performed. NMR spectroscopy (Figure S2) demonstrates that in the dissected sciatic nerve, the only fluorine signature arises from the ^19^F in the PFPE.

The ^19^F signal localizes at the site of sciatic nerve injury in the CCI rats and no such signal was observed in the sham rat (Figure 4). The presence of ^19^F signal was also observed in the femur and tibia of CCI and sham rats. Complex regional pain syndrome has previously been associated with osteoporotic changes in human subjects [58]. Furthermore, it has been demonstrated that osteoporotic changes in the ipsilateral tibial bone occur after CCI surgery. These changes are also associated with an increased number of osteoclasts in the bone [59]. Hence, the ^19^F signal we observed in the bone marrow (Figure 4) could be indicative of the osteoporotic changes in the femur and tibia of CCI rats and is a result of the peripheral nerve injury. It has also been shown that a subset of blood monocytes could serve as osteoclast precursors [60]. Overall, our interpretation is that the ^19^F signal evident in the bone marrow is due to migration of labeled monocytes to the bone.

Immunohistochemistry performed on the ipsilateral sciatic nerve of CCI rat clearly demonstrates infiltration of macrophages in the sciatic nerve (Figure 5). No such infiltration was observed for the left leg contralateral to the CCI, the ipsilateral sham and the control nerves (Figure 5). Therefore, the ^19^F signal and the fluorescence observed during *in vivo* and *ex vivo* NIR imaging is due to infiltration of labeled macrophages at the site of injury.

## Conclusion

This study demonstrates that the infiltration of immune cells into the CCI affected sciatic nerve can be monitored in live animals. Furthermore, the neuroinflammation caused by the CCI surgery can be imaged using the dual mode nanoemulsion such that both live animal NIR imaging and post-mortem perfluorocarbon ^19^F MRI generate complementary results demonstrating that this neuroinflammation contributes to the pain associated with CCI surgery. In rats, specifically targeting the immune cells *in vivo* may serve as a treatment for neuroinflammation. The same technique could be used to monitor the effect of therapeutic drugs by assessing the extent of inflammation at the site of injury and to diagnose neuroinflammation without the need for surgical intervention involving a nerve biopsy.

## Supporting Information

Figure S1 **Near Infrared imaging (NIR) of live rats merged with white light images with the Region of Interest (ROI) indicated.** All of these images were acquired on the LiCOR Pearl Live animal imager and processed in the same linked look-up tables. The ROI was set for the right leg CCI condition and then copied to a similar relative position on each of other legs for that experimental set. The LiCOR software tabulates the relative fluorescent signal and area (note Table S2).
(TIF)

Figure S2 **^19^F NMR spectra for nanoemulsion in dissected CCI sciatic nerve.** Exhibits ^19^F NMR spectrum of dissected right sciatic nerve from CCI animal. TFA (−76.00 ppm) reference standard is shown along with the tissue signal for the nanoemulsion in the sciatic nerve (−92.5 ppm).
(TIFF)

Figure S3 **Near Infrared imaging (NIR) of live rats merged with white light images of their body, 2 days post intravenous injection of labeled emulsion on day 11 post surgery.** All of these images were acquired on the LiCOR Pearl Live animal imager and processed in the same experiment with linked look-up tables. The control, sham and CCI (**A–F**) exhibit auto fluorescence over the thoracolumbar region, which is also evident prior to injection (Note Figure 2). The control animal (**A**) does not exhibit any fluorescence over the right leg. The sham (**B**) similarly does not exhibit any fluorescence in the area of the surgical wound (surgical staples). The CCI animal (**C**) exhibits a wide area of fluorescent signal over much of the thigh. The left side of the control, sham and CCI (**D, E, F**) exhibit only the auto fluorescence in the thoracolumbar region. Bar = 1 cm.
(TIFF)

Table S1 **Primer Sequences used for this study.**
(DOCX)

Table S2 **LiCOR total relative fluorescence for each ROI.** The Mean (Total Relative Fluorescence/Area) for each ROI is used for the analysis of variance (ANOVA) and t-test.
(DOCX)

Table S3 **1 Way ANOVA of Relative NIR Fluorescence in Live Rats.**
(DOCX)
