# Supplementary figures and images for: Imaging Neuroinflammation In Vivo in a Neuropathic Pain Rat Model with Near-Infrared Fluorescence and 19F Magnetic Resonance
Source: PLoS One. 2014 Feb 28;9(2):e90589. doi: 10.1371/journal.pone.0090589 (PMC3938771; doi:10.1371/journal.pone.0090589)

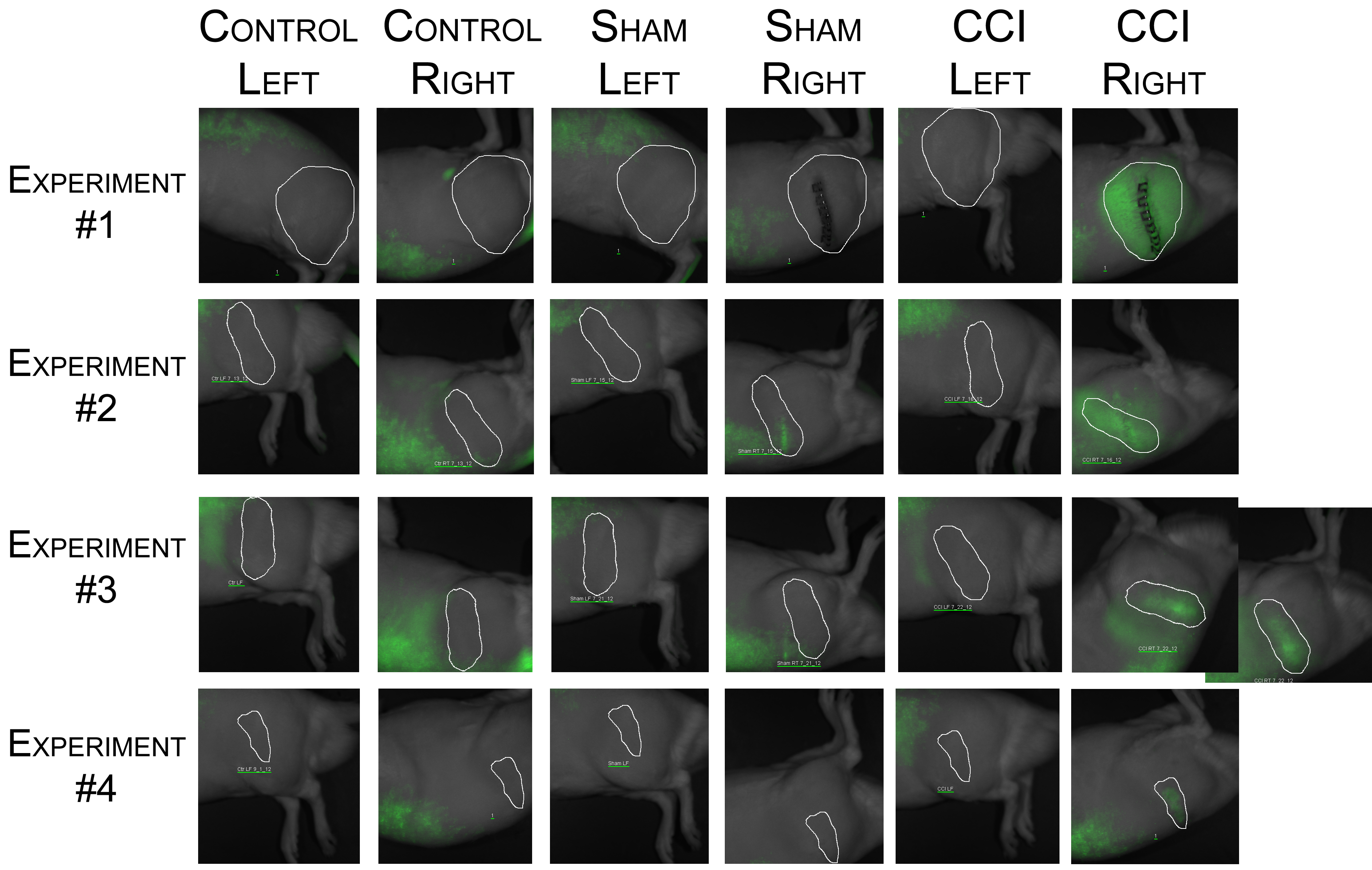

Supplement: Figure S1 — Near Infrared imaging (NIR) of live rats merged with white light images with the Region of Interest (ROI) indicated. All of these images were acquired on the LiCOR Pearl Live animal imager and processed in the same linked look-up tables. The ROI was set for the right leg CCI condition and then copied to a similar relative position on each of other legs for that experimental set. The LiCOR software tabulates the relative fluorescent signal and area (note Table S2). (TIF) [file pone.0090589.s001.tif]

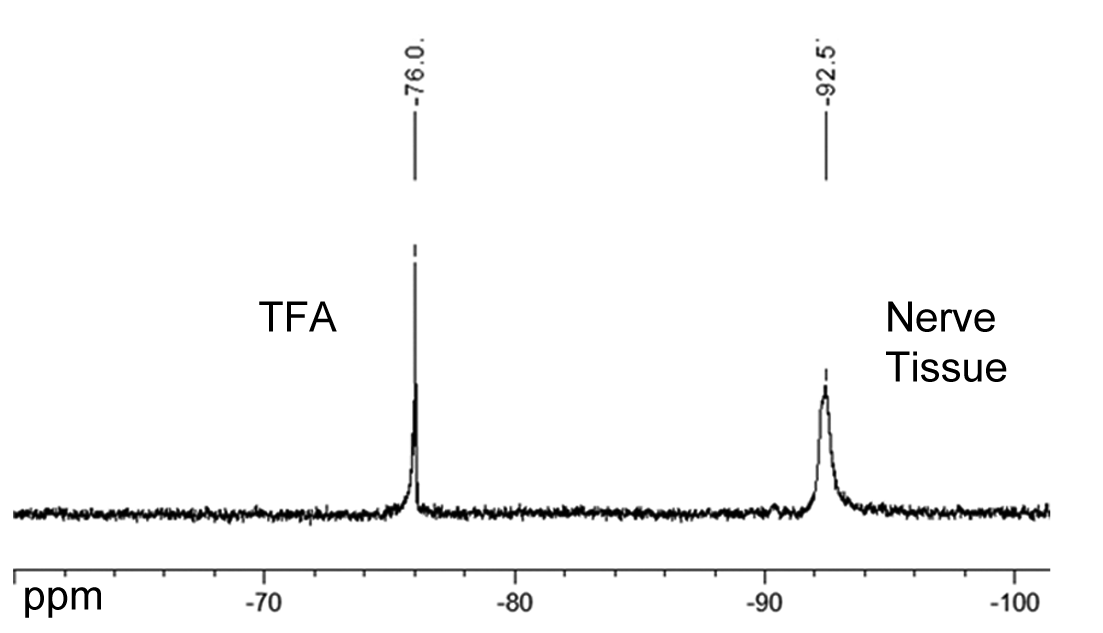

Supplement: Figure S2 — 19F NMR spectra for nanoemulsion in dissected CCI sciatic nerve. Exhibits 19F NMR spectrum of dissected right sciatic nerve from CCI animal. TFA (−76.00 ppm) reference standard is shown along with the tissue signal for the nanoemulsion in the sciatic nerve (−92.5 ppm). (TIFF) [file pone.0090589.s002.tiff]

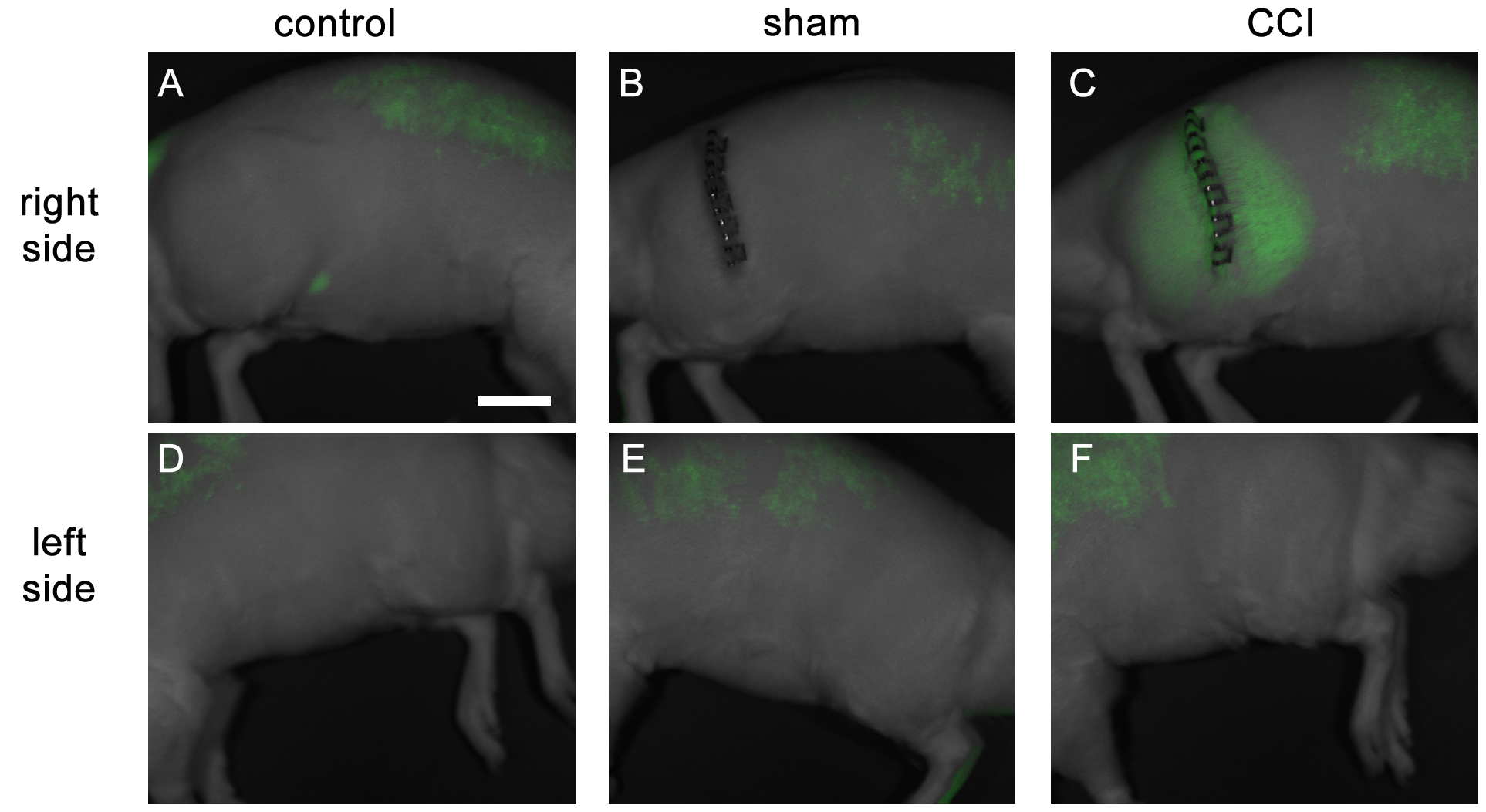

Supplement: Figure S3 — Near Infrared imaging (NIR) of live rats merged with white light images of their body, 2 days post intravenous injection of labeled emulsion on day 11 post surgery. All of these images were acquired on the LiCOR Pearl Live animal imager and processed in the same experiment with linked look-up tables. The control, sham and CCI (A–F) exhibit auto fluorescence over the thoracolumbar region, which is also evident prior to injection (Note Figure 2). The control animal (A) does not exhibit any fluorescence over the right leg. The sham (B) similarly does not exhibit any fluorescence in the area of the surgical wound (surgical staples). The CCI animal (C) exhibits a wide area of fluorescent signal over much of the thigh. The left side of the control, sham and CCI (D, E, F) exhibit only the auto fluorescence in the thoracolumbar region. Bar = 1 cm. (TIFF) [file pone.0090589.s003.tiff]
