# Supplementary material for: Imaging Neuroinflammation In Vivo in a Neuropathic Pain Rat Model with Near-Infrared Fluorescence and 19F Magnetic Resonance
Source: PLoS One. 2014 Feb 28;9(2):e90589. doi: 10.1371/journal.pone.0090589 (PMC3938771; doi:10.1371/journal.pone.0090589)
Supplement: Table S1 — Primer Sequences used for this study. (DOCX) [file pone.0090589.s004.docx]

Supplemental Table 1. Primer Sequences used for this study.

| **GAPDH** |  |  |  |  |
| --- | --- | --- | --- | --- |
|  | Tm (°C) | GC content (%) | Sequence (5’-3’) | Amplicon length |
| Forward | 55.4 | 55.0 | CACAGTCAAGGCTGAGAATGG |  |
| Reverse | 54.7 | 55.0 | CGATGCCAAAGTTGTCATGG |  |
|  |  |  |  | 300bp |
| **TRPV1** |  |  |  |  |
|  | Tm (°C) | GC content (%) | Sequence | Amplicon length |
| *Forward | 56.2 | 55.0 | GTGATCGCTTACAGCAGCAG |  |
| *Reverse | 55.0 | 55.0 | CGGTGACTCGGAAATAGTCC |  |
|  |  |  |  | 238bp |
| **NPY** |  |  |  |  |
|  | Tm (°C) | GC content (%) | Sequence | Amplicon length |
| Forward | 49.6 | 47.4 | GACAGAGATATGGCAAGAG |  |
| Reverse | 48.9 | 47.4 | CTAGGAAAAGTCAGGAGAG |  |
|  |  |  |  | 148bp |
| **Gap43** |  |  |  |  |
|  | Tm (°C) | GC content (%) | Sequence | Amplicon length |
| Forward | 51.0 | 50.0 | CCTAAACAAGCCGATGTG |  |
| Reverse | 49.7 | 44.0 | TTTGGCTTCATCTACAGC |  |
|  |  |  |  | 150bp |

* primer sequences acquired from Charrua A, Reguenga C, Paule CC, Nagy I, Cruz F, Avelino A. (2008). Cystitis is associated with TRPV1b-downregulation in rat dorsal root ganglia. Neuroreport 19(15):1469-1472.
