# Supplementary material for: Imaging Neuroinflammation In Vivo in a Neuropathic Pain Rat Model with Near-Infrared Fluorescence and 19F Magnetic Resonance
Source: PLoS One. 2014 Feb 28;9(2):e90589. doi: 10.1371/journal.pone.0090589 (PMC3938771; doi:10.1371/journal.pone.0090589)
Supplement: Table S2 — LiCOR total relative fluorescence for each ROI. The Mean (Total Relative Fluorescence/Area) for each ROI is used for the analysis of variance (ANOVA) and t-test. (DOCX) [file pone.0090589.s005.docx]

**Table S2. LiCOR total relative fluorescence for each ROI.** The Mean (Total Relative Fluorescence/Area) for each ROI is used for the analysis of variance (ANOVA) and t test.

|  | **Image ID** | **Name** | **Polygon** | **Total** | **Mean**  **(total/area)** | **Std. Dev.** | **Min** | **Max** | **Area (pixels)** |
| --- | --- | --- | --- | --- | --- | --- | --- | --- | --- |
|  |  |  |  |  |  |  |  |  |  |
| Experiment #1 | 0000331_02 | Ctr Right | 1 | 2.72E2 | 6.28E-3 | 2.02E-3 | 1.45E-3 | 1.95E-2 | 43369 |
|  | 0000333_02 | Ctr Left | 1 | 2.46E2 | 5.67E-3 | 2.22E-3 | 1.05E-3 | 2.14E-2 | 43369 |
|  | 0000335_02 | Sham Right | 1 | 3.78E2 | 8.73E-3 | 4.22E-3 | 5.50E-4 | 3.31E-2 | 43369 |
|  | 0000337_02 | Sham Left | 1 | 4.78E2 | 1.10E-2 | 5.79E-3 | 1.95E-3 | 3.49E-2 | 43369 |
|  | 0000348_02 | CCI Right | 1 | 1.75E3 | 4.05E-2 | 1.18E-2 | 1.80E-3 | 7.41E-2 | 43369 |
|  | 0000349_02 | CCI Left | 1 | 3.95E2 | 9.10E-3 | 3.44E-3 | 1.30E-3 | 2.68E-2 | 43369 |
| Experiment #2 | 0000454_02 | Ctr Right | 1 | 3.04E2 | 1.84E-2 | 4.78E-3 | 9.60E-3 | 4.14E-2 | 13271 |
|  | 0000455_02 | Ctr Left | Ctr LF | 2.34E2 | 1.42E-2 | 4.34E-3 | 6.00E-3 | 3.53E-2 | 12347 |
|  | 0000460_02 | Sham Right | ShamRT | 3.75E2 | 2.28E-2 | 8.34E-3 | 9.80E-3 | 5.91E-2 | 12653 |
|  | 0000461_02 | Sham Left | ShamLF | 1.92E2 | 1.17E-2 | 4.41E-3 | 5.10E-3 | 3.39E-2 | 13739 |
|  | 0000464_02 | CCI Right | CCI RT | 7.21E2 | 4.37E-2 | 6.77E-3 | 2.02E-2 | 6.43E-2 | 16486 |
|  | 0000466_02 | CCI Left | CCI LF | 1.94E2 | 1.18E-2 | 3.35E-3 | 4.65E-3 | 3.07E-2 | 11751 |
| Experiment #3 | 0000470_02 | Ctr Right | Ctr RT | 3.71E2 | 2.15E-2 | 4.24E-3 | 1.24E-2 | 3.88E-2 | 16498 |
|  | 0000471_02 | Ctr Left | Ctr LF | 3.24E2 | 1.88E-2 | 3.81E-3 | 9.85E-3 | 3.63E-2 | 16478 |
|  | 0000478_02 | Sham Right | ShamRT | 2.66E2 | 1.55E-2 | 4.73E-3 | 7.55E-3 | 4.25E-2 | 16798 |
|  | 0000479_02 | Sham Left | ShamLF | 2.62E2 | 1.52E-2 | 4.29E-3 | 8.00E-3 | 4.06E-2 | 16489 |
|  | 0000487_02 | CCI Right | CCI RT | 5.87E2 | 3.41E-2 | 8.07E-3 | 1.30E-2 | 6.74E-2 | 13708 |
|  | 0000488_02 | CCI Right | CCI RT | 5.72E2 | 3.32E-2 | 7.12E-3 | 1.30E-2 | 6.14E-2 | 17210 |
|  | 0000490_02 | CCI Left | CCI LF | 2.22E2 | 1.29E-2 | 2.44E-3 | 5.90E-3 | 2.31E-2 | 17210 |
| Experiment #4 | 0000715_02 | Ctr Left | Ctr LF | 5.54E1 | 8.08E-3 | 1.74E-3 | 3.20E-3 | 1.64E-2 | 6861 |
|  | 0000718_04 | Ctr Right | 1 | 6.70E1 | 9.76E-3 | 1.92E-3 | 4.85E-3 | 1.91E-2 | 6861 |
|  | 0000725_02 | Sham Left | ShamLF | 4.38E1 | 6.39E-3 | 3.01E-3 | 7.50E-4 | 1.64E-2 | 6861 |
|  | 0000727_02 | Sham Right | ShamRT | 4.31E1 | 6.28E-3 | 1.45E-3 | 2.00E-3 | 1.22E-2 | 6861 |
|  | 0000739_02 | CCI Left | CCI LF | 8.01E1 | 1.17E-2 | 4.33E-3 | 4.05E-3 | 2.47E-2 | 6861 |
|  | 0000742_02 | CCI Right | 1 | 1.65E2 | 2.41E-2 | 7.35E-3 | 2.05E-3 | 4.40E-2 | 6861 |
