# Supplementary material for: Imaging Neuroinflammation In Vivo in a Neuropathic Pain Rat Model with Near-Infrared Fluorescence and 19F Magnetic Resonance
Source: PLoS One. 2014 Feb 28;9(2):e90589. doi: 10.1371/journal.pone.0090589 (PMC3938771; doi:10.1371/journal.pone.0090589)
Supplement: Table S3 — 1 Way ANOVA of Relative NIR Fluorescence in Live Rats. (DOCX) [file pone.0090589.s006.docx]

**Table S3.** **1 Way ANOVA of Relative NIR Fluorescence in Live Rats.**

|  | Naïve  Left | Naïve  Right | Sham  Left | Sham  Right | CCI  Left | ***  CCI  Right | |
| --- | --- | --- | --- | --- | --- | --- | --- |
| Expt #1 | 0.0057 | 0.0063 | 0.0110 | 0.0087 | 0.0091 | 0.041 | |
| Expt #2 | 0.0140 | 0.0180 | 0.0120 | 0.0230 | 0.0120 | 0.044 | |
| Expt #3 | 0.0190 | 0.0220 | 0.0150 | 0.0160 | 0.0130 | 0.034 | 0.033 |
| Expt #4 | 0.0080 | 0.0098 | 0.0064 | 0.0063 | 0.0120 | 0.024 | |
|  |  |  |  |  |  |  | |
| n | 4 | 4 | 4 | 4 | 4 | 5 | |
| Mean | 0.01168 | 0.0140 | 0.0111 | 0.0135 | 0.01153 | 0.0352 | |
| Std. Deviation | 0.0060 | 0.0073 | 0.0036 | 0.0076 | 0.0017 | 0.0078 | |
| Std. Error | 0.0030 | 0.0036 | 0.0018 | 0.0038 | 0.0008 | 0.0035 | |
|  |  |  |  |  |  | **p <0.0001** | |

t test of Sham Right versus CCI Right p value <0.0001
